# Dynamic Modeling of Fouling in Reverse Osmosis Membranes

**DOI:** 10.3390/membranes11050349

**Published:** 2021-05-10

**Authors:** Bowen Ling, Peng Xie, David Ladner, Ilenia Battiato

**Affiliations:** Institute of Mechanics, Chinese Academy of Sciences, Beijing 100190, China; lingbowen@imech.ac.cn (B.L.); pxie@g.clemson.edu (P.X.); ladner@clemson.edu (D.L.)

**Keywords:** RO membrane, numerical model, OpenFoam

## Abstract

During reverse osmosis (RO) membrane filtration, performance is dramatically affected by fouling, which concurrently decreases the permeate flux while increasing the energy required to operate the system. Comprehensive design and optimization of RO systems are best served by an understanding of the coupling between membrane shape, local flow field, and fouling; however, current studies focus exclusively on simplified steady-state models that ignore the dynamic coupling between fluid flow, solute transport, and foulant accumulation. We developed a customized solver (SUMs: Stanford University Membrane Solver) under the open source finite volume simulator OpenFOAM to solve transient Navier–Stokes, advection–diffusion, and adsorption–desorption equations for foulant accumulation. We implemented two permeate flux reduction models at the membrane boundary: the resistance-in-series (RIS) model and the effective-pressure-drop (EPD) model. The two models were validated against filtration experiments by comparing the equilibrium flux, pressure drop, and fouling pattern on the membrane. Both models not only predict macroscopic quantities (e.g., permeate flux and pressure drop) but also the fouling pattern developed on the membrane, with a good match with experimental results. Furthermore, the models capture the temporal evolution of foulant accumulation and its coupling with flux reduction.

## 1. Introduction

Reverse osmosis (RO) filtration systems are widely applied in seawater desalination [1,2,3,4,5], landfill leachate treatment [6,7], and wastewater reclamation [8,9,10,11,12,13,14]. Typically, RO performs one of the final stages of water treatment and is designed to remove ions or soluble substances.

Due to the extraction of the solvent (e.g., water) on the feed side close to the membrane surface, the solute concentration rises, which is known as concentration polarization (CP) [15,16,17,18,19]. Some solutes can precipitate or crystallize on the membrane surface, while other solutes adsorb to the membrane, hindering permeation of the solvent and reducing the efficiency of the membrane [20,21]. Such fouling processes cause reduction of clean water permeate flux. By increasing the applied pressure, one can increase the pressure gradient across the membrane to force a larger permeate flux, but the energy input per unit flux increases as a result. Fouling depends on the solute and membrane properties; for instance, biologically active foulants can produce thick, relatively low permeability biofilms [22]. RO membrane modules require spacers to separate membrane leaves and create flow channels; these spacers play an important role in fouling development. For example, the most commonly used net-like spacers create dead zones where foulant cake growth is accentuated [23].

Different mechanisms can be utilized at different scales to control fouling. These include: (i) changes in the morphology (shape) of the flow channel at the system scale (∼cm) [24,25,26,27,28]; (ii) modifications to the topology of the membrane surface at the micro-scale (∼mm–μm) [29,30,31,32,33,34,35]; (iii) chemical or surface treatment which changes the interaction force between foulant and membrane at the nano-scale (∼nm) [36,37,38].

It has been shown that morphological changes can provide in-situ fouling mitigation; a number of studies [35,39,40,41] have demonstrated that flow and solute transport at both the macro- and micro-scales can be controlled by modifying the membrane/spacer morphology. However, most analyses still optimize the system by trial and error since a general framework to study foulant deposition and in situ control is still not available. Due to experimental difficulties and cost, performing extensive studies on different configurations is challenging.

Computational models [5,25,42,43] represent an attractive alternative to more expensive experimentation as they allow one to virtually span the entire design space at a fraction of the cost; however, foulant dynamical behavior is elusive for most existing models. The major challenges associated with modeling dynamic fouling processes are (i) the temporal evolution of foulant deposition and (ii) the strong coupling between flow, bulk solute concentration, and foulant deposition. Complex spacer geometry complicates the matter even further, and, while inherently essential to RO system optimization, modeling the spatio-temporal evolution of fouling remains an open challenge.

Most of the models that account for the temporal evolution of the foulant layer do so without a full coupling between flow, transport, and foulant deposition. For example, Bucs et al. [22] model the thickness of foulant as an empirically postulated function of time with a constant growth rate, yet the velocity and bulk concentration fields are determined from steady-state equations. Xie et al. [44] model fouling accumulation using a temporal adsorption/desorption equation under the hypothesis that the adsorption rate depends on the local bulk concentration. The authors also introduce in their model the process of mechanical removal of foulant due to hydrodynamic shear by introducing a stress term into the growth equation for the foulant. Again, not only are the flow and concentration fields solved by steady-state equations, but the flow field is imposed as a background field without accounting for the feedback from fouling processes. Lyster and Cohen [45] propose a set of equations and boundary conditions that couple the velocity component orthogonal to the membrane surface with the local concentration gradient on the membrane surface. While these conditions, derived by mass balance in two dimensions, are shown to successfully capture concentration polarization (CP) and the coupling between CP, flow and bulk concentration, the model does not account for unsteady terms and does not include a mechanism to relate CP to fouling.

Recently, Ling and Battiato [46] developed a model that couples the transient Navier–Stokes and the advection–diffusion equations, as well as an adsorption–desorption equation for foulant accumulation. Although they validate it against experimental data and demonstrate that it is able to correctly capture unsteady measurements of permeate flux, its capability of correctly capturing spatial distribution of the foulant in morphologically complex membranes was not evaluated. This is a critical step in assessing the potential of using the model as a virtual laboratory for design and membrane performance optimization purposes. It is worth noticing that Ling and Battiato used an effective-pressure-drop (EPD) model, which couples the flux reduction and fouling accumulation by introducing an additional pressure reducing term. The EPD model varies from the more classical approach of treating the foulant layer as an additional flow resistance, which is often referred to as a resistance-in-series (RIS) model [47]. In this study, we use both approaches and compare them.

Here, all processes are modeled using a 3D fully-coupled system of transient equations: the Navier–Stokes equations for flow, an advection–diffusion equation for the bulk concentration, and an adsorption–desorption equation for fouling. Furthermore, the model allows one to relate concentration polarization, occurring in the bulk solution, with fouling taking place on the membrane modeled as a surface concentration. The flux reduction induced by foulant accumulation is modeled using an adsorption–desorption equation which associates the local bulk concentration, foulant surface concentration, and permeate flux. All equations are implemented through a customized solver SUMS (Stanford University Membrane solver) in the open-source finite-volume framework OpenFoam.

The model is validated by comparing three-dimensional simulations with fouling experiments conducted by Xie and et al. [44], who measured (i) the permeate flux and pressure drop and (ii) the spatial distribution of fouling patterns for different spacer configurations. Such comparisons demonstrate the RIS and EPD models’ capability of capturing both system-scale quantities (i.e., flux and pressure) and local effects (fouling pattern). The spacers studied by Xie et al. do not have conventional geometry; they comprise a set of sinusoidal flow channels that vary in amplitude and frequency. This experimental data set, with its unique design, has a wider variation in geometry (and thus a wider range of flow patterns) and spatial scales than most spacer studies; in addition, the data were readily available to us in raw form, making this a useful data set for testing the effectiveness of the SUMS framework.

The paper is organized with Section 2 introducing the governing equations and simulation scenarios. In Section 3, we present the experimental setup and data post-processing technique to digitize the images of fouling patterns. In Section 4, we compare the simulated permeate flux, pressure drop, and fouling pattern with the corresponding experimental results. We provide concluding remarks in Section 5.

## 2. Materials and Methods

### 2.1. Formulation

**Figure 1:**
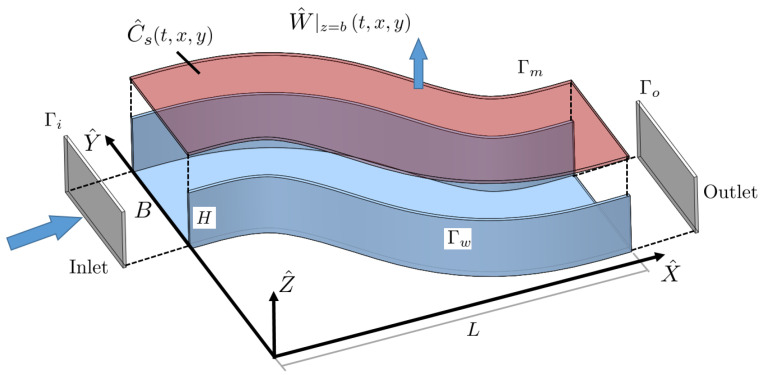
Three-dimensional sketch of the domain, with the definition of wall and membrane surfaces.

We are interested in studying fouling accumulation on a flat sheet membrane as a function of time, $\hat{T}$, and location, $\hat{X}=\left(\hat{X},\hat{Y},\hat{Z}\right)$. The flow field ($\hat{U}$) of an incompressible viscous fluid satisfies the Navier–Stokes and continuity equations

(1a) $$\begin{array}{cc} \; & \frac{\partial \hat{U}}{\partial \hat{T}}+\left(\hat{U}\cdot \hat{\nabla }\right)\hat{U}+\frac{1}{\rho }\hat{\nabla }\hat{P}=\hat{\nabla }\cdot \left(\nu \hat{\nabla }\hat{U}\right) \end{array}$$

(1b) $$\begin{array}{cc} \; & \hat{\nabla }\cdot \hat{U}=0 \end{array}$$

where $\hat{U}$ [m/s] is a three-dimensional velocity field $\hat{U}=\left(\hat{U},\hat{V},\hat{W}\right)$, $\hat{P}$ [kg m^−1^s^−2^] is the pressure, ρ [kg/m^3^] is the fluid density, and ν [m^2^/s] is the fluid kinematic viscosity. Gravity is neglected in this study. The solute bulk concentration satisfies an advection–diffusion equation

(2) $$\begin{array}{cc} \; & \frac{\partial {\hat{C}}_{b}}{\partial \hat{T}}+\hat{U}\cdot \hat{\nabla }{\hat{C}}_{b}-D{\hat{\nabla }}^{2}{\hat{C}}_{b}=0, \end{array}$$

where ${\hat{C}}_{b}\left(\hat{X},\hat{T}\right)$ is the solute bulk concentration [mol/m^3^] in the liquid domain, and *D* [m^2^/s] is the molecular diffusion coefficient of the solute in water. A Langmuir adsorption–desorption equation (defined on the membrane surface) is used to model foulant accumulation on the membrane located at $\hat{Z}=H$ (see Figure 1, i.e., the surface concentration of the foulant ${\hat{C}}_{s}$ [mol/m^2^], defined at $\hat{Z}=H$, satisfies

(3) $$\begin{array}{c} \frac{\partial {\hat{C}}_{s}}{\partial \hat{T}}=K_{1}\left({\hat{C}}_{s,\max}-{\hat{C}}_{s}\right)\hat{C}-K_{2}{\hat{C}}_{s}, \end{array}$$

where $K_{1}$ [1/(mol·s)] is the adsorption coefficient, $K_{2}$ [1/s] is the desorption coefficient and ${\hat{C}}_{s,\max}$ is the equilibrium foulant concentration. The adsorption model uses the liquid–domain concentration adjacent to the membrane, ${\hat{C}}_{b}$, to determine the driving force for foulant adsorption on the membrane. It is worth emphasizing that the same kinetic equation has been adopted in both organic foulant growth [44,48] and crystal growth [49] modeling, where $K_{1}$ and $K_{2}$ can be determined via experiments. Additionally, such a framework allows one to evaluate concentration polarization and foulant accumulation individually. Ion (e.g., Ca${}^{2+}$) transport in solution is modeled by ${\hat{C}}_{b}$ and its crystallization (e.g., CaSO${}_{4}$ or CaCO${}_{3}$) and accumulation on the membrane is modeled by ${\hat{C}}_{s}$.

The previous equations are supported by appropriate boundary conditions at the inlet and outlet for the momentum and mass transport problems. Specifically,

(4) $$\begin{array}{c} \hat{U}\left(\hat{X}=0,\hat{Y},\hat{Z}\right)=U_{\mathrm{in}}\;\mathrm{and}\;\hat{C}\left(\hat{X}=0,\hat{Y},\hat{Z}\right)=C_{0} \end{array}$$

On the solid walls of the channel, no-slip and no-penetration conditions are employed. On the membrane surface, the velocity components $\hat{U}$ and $\hat{V}$ are modeled by the Beavers–Joseph condition [50],

(5) $$\begin{array}{cc} \hat{U}= & \frac{\sqrt{{\hat{K}}_{m}}}{\hat{\beta }}\frac{\partial \hat{U}}{\partial \hat{Z}}, \end{array}$$

(6) $$\begin{array}{cc} \hat{V}= & \frac{\sqrt{{\hat{K}}_{m}}}{\hat{\beta }}\frac{\partial \hat{V}}{\partial \hat{Z}}, \end{array}$$

where $\hat{\beta }$ is a constant that only depends on the geometry of the membrane porous structure. In addition, the flux balancing boundary condition proposed by Lyster and Cohen [45]

(7) $$\frac{\partial {\hat{C}}_{b}}{\partial \hat{Z}}=-\frac{R_{i}}{D}\hat{W}{\hat{C}}_{b}$$

is employed. In (7), $\hat{W}$ is the permeate water flux, $R_{i}$ is the intrinsic membrane rejection rate [45], (set to $R_{i}=100\%$ in this study). The permeate flux across a clean membrane is modeled as:

(8) $$\hat{W}={\hat{K}}_{m}\left(\Delta \hat{P}-\Delta \hat{\Pi }\right),$$

where ${\hat{K}}_{m}$ is the hydraulic membrane water permeability in the absence of fouling (i.e., when ${\hat{C}}_{s}=0$), the pressure drop $\Delta \hat{P}$ is defined as $\Delta \hat{P}=\hat{P}-P_{\mathrm{amb}}$, with $\hat{P}$ the local pressure and $P_{\mathrm{amb}}$ the ambient pressure, here set to zero. $\Delta \hat{\Pi }$ is the osmotic pressure difference between the feed and permeate, here we assume concentration at the permeate side is zero, namely:

(9) $$\hat{W}={\hat{K}}_{m}\left(\Delta \hat{P}-{\hat{A}}_{o}{\hat{C}}_{b}{|}_{Z=H}\right),$$

where ${\hat{A}}_{o}$ [$m^{2}/\left(s\cdot \mathrm{mol}\right)$] is the osmotic coefficient and ${\hat{C}}_{b}{|}_{Z=H}$ is the bulk concentration near the membrane surface. When the local concentration increases, the permeate flux decreases due to the osmotic pressure. Additional flux reduction due to fouling can be modeled through (i) a resistance-in-series (RIS) model, and (ii) an effective pressure drop (EPD) model, which are discussed in the following.

### 2.2. Resistance-in-Series Model

The RIS model treats the foulant layer and the membrane as flow resistors that connect in series such that the fouled membrane permeability is ${\hat{K}}_{\mathrm{eff}}\left({\hat{C}}_{s}\right)$ and is modeled as

(10) $${\hat{K}}_{\mathrm{eff}}=\frac{1}{R_{m}+R_{f}}.$$

The former relationship quantifies the combined resistance induced by the membrane and the accumulated foulant. In (10), $R_{m}$ is the clean membrane resistance,

(11) $$R_{m}=\frac{1}{{\hat{K}}_{m}},$$

and $R_{f}$ is the fouled membrane resistance,

(12) $$R_{f}=\frac{C_{s}}{{\hat{K}}_{f}},$$

where ${\hat{K}}_{f}$ is the fouled membrane permeability and $C_{s}$ is the normalized surface concentration: $C_{s}={\hat{C}}_{s}/{\hat{C}}_{s,\max}$. When $C_{s}=1$, the foulant layer results in the maximum flow resistance. The foulant permeability ${\hat{K}}_{f}$ is modeled as a proportion of the clean membrane permeability, i.e.,

(13) $${\hat{K}}_{f}=A_{k}C_{s}{\hat{K}}_{m},$$

where $A_{k}=\left(0,1\right]$ is a dimensionless constant. Combining (9) with (10), while accounting for (11)–(13), the permeate flux across a fouled membrane in the RIS model can be written as

(14) $${\hat{W}}_{\mathrm{RIS}}=\frac{\Delta \hat{P}-{\hat{A}}_{o}{\hat{C}}_{b}}{R_{m}\left(1+C_{s}/A_{k}\right)}=\frac{A_{k}{\hat{K}}_{m}}{C_{s}+A_{k}}\left(\Delta \hat{P}-{\hat{A}}_{o}{\hat{C}}_{b}\right).$$

It is worth emphasizing that, when $C_{s}=0$, then ${\hat{K}}_{\mathrm{eff}}={\hat{K}}_{m}$, then relationship (9) for clean membranes is recovered. However, the model cannot capture local clogging of the membrane (i.e., $W_{\mathrm{RIS}}=0$) when $C_{s}=1$, since such condition would require $R_{f}\to \infty$, or $A_{k}\left(C_{s}\right)$ such that $A_{k}\left(C_{s}=1\right)=0$, which contradicts the model formulation where $A_{k}$ is just a fitting constant different from zero.

### 2.3. Effective Pressure Drop Model

In the EPD model, Equation (9) is generalized under fouled conditions through a modification of the effective driving pressure drop, $\left(\Delta \hat{P}-{\hat{A}}_{o}{\hat{C}}_{b}\right)$, where a pressure reduction due to local foulant accumulation, ${\hat{A}}_{p}{\hat{C}}_{s}$ [46], is introduced,

(15) $${\hat{W}}_{\mathrm{EPD}}={\hat{K}}_{m}\left(\Delta \hat{P}-{\hat{A}}_{o}{\hat{C}}_{b}-{\hat{A}}_{p}{\hat{C}}_{s}\right).$$

In (15), ${\hat{A}}_{p}$ is a foulant coefficient. Equation (9) for clean membranes is readily recovered when ${\hat{C}}_{s}=0$ and ${\hat{C}}_{b}=0$. In addition, (15) is able to capture local blockage (${\hat{W}}_{\mathrm{EPD}}=0$) when ${\hat{A}}_{o}{\hat{C}}_{b}+{\hat{A}}_{p}=\Delta \hat{P}$. Additionally, the EPD formulation (15) directly associates the flux reduction with precipitation kinetics. This allows one to achieve the coupling between flow, bulk transport, and foulant deposition exclusively through boundary conditions on the membrane surface, without the need for additional ad hoc parametrization of the fouled membrane resistance. In this study, we will compare these two approaches.

Once the transient, coupled flow and transport problems are solved by using the RIS or the EPD model for fouling, the permeate flow rate $\hat{Q}$ $\left[m^{3}/s\right]$ can be calculated as

(16) $$\hat{Q}=\frac{\int_{\Gamma_{n}}{\hat{W}}_{i}dA}{\int_{\Gamma_{m}}dA},\;i=\left\{\mathrm{RIS},\mathrm{EPD}\right\}$$

where $\hat{W}$ is defined by either (14) or (15), respectively, and $\Gamma_{n}$ is the non-fouled region of the membrane surface and is defined by using a threshold value of the surface concentration $C_{s}$, i.e., $\alpha C_{s,\max}$ (with α=0.7 in this study), as

(17) $$\Gamma_{n}\in \left\{\Gamma_{m}|C_{s}\geq \alpha C_{s,\max},\alpha \in \left[0,1\right]\right\}.$$

The set of Equations (1)–(17) can be cast in dimensionless form. We define the dimensionless quantities

(18) $$\begin{array}{c} u=\frac{\hat{U}}{U_{\mathrm{in}}},\;\;x=\frac{\hat{X}}{B},\;\;t=\frac{U_{\mathrm{in}}\hat{T}}{B},\;\;P=\frac{B^{2}\hat{P}}{\nu^{2}},\;\;h=\frac{H}{B},\;\;C_{s}=\frac{{\hat{C}}_{s}}{C_{s,\max}},\;\;C_{b}=\frac{\hat{C}}{C_{0}}, \end{array}$$

where u=(u,v,w) and x=(x,y,z) are the dimensionless velocity field and coordinate axes, respectively. We also introduce the following dimensionless numbers,

(19) Re=UinBν,Pe=UinBD,DaI=K1BC0Uin,DaII=K2BUin,

where Re, Pe, ${\mathrm{Da}}_{i}$, i={I,II} are the Reynolds, Péclet and Damköhler numbers, respectively. Then, the dimensionless form of Equations (1)–(3) reads as follows:

(20a) ∂u∂t+(u·∇)u+∇P=1Re∇2u,

(20b) $$\begin{array}{cc} \; & \nabla \cdot u=0, \end{array}$$

for flow, and

(21a) $$\begin{array}{cc} \; & \mathrm{Pe}\left(\frac{\partial C_{b}}{\partial t}+u\cdot \nabla C_{b}\right)-\nabla^{2}C_{b}=0, \end{array}$$

(21b) $$\begin{array}{cc} \; & \frac{\partial C_{s}}{\partial t}={\mathrm{Da}}_{I}\left(1-C_{s}\right)C-{\mathrm{Da}}_{\mathrm{II}}C_{s}, \end{array}$$

for transport. On the membrane surface (z=h), the dimensionless boundary conditions for flow and transport are slip conditions in the direction parallel to the membrane

(22) $$\begin{array}{cc} u_{h}= & \frac{\sqrt{k_{m}}}{\beta }\frac{\partial u_{h}}{\partial z} \end{array}$$

(23) $$\begin{array}{cc} v_{h}= & \frac{\sqrt{k_{m}}}{\beta }\frac{\partial v_{h}}{\partial z} \end{array}$$

where β is the Beavers–Joseph constant and is selected to be 2, and the dimensionless flux balancing condition for mass transport

(24) $$\begin{array}{c} \frac{\partial C_{b}}{\partial z}=\mathrm{Pe}\;w_{i}C_{b},\;i=\left\{\mathrm{RIS},\mathrm{EPD}\right\} \end{array}$$

where

(25) $$\begin{array}{c} A_{o}=\frac{{\hat{A}}_{o}B^{2}}{\nu^{2}},\;w_{h}=\frac{W_{H}}{U_{\mathrm{in}}},\;\;\Delta P=\frac{\Delta \hat{P}B^{2}}{\nu^{2}}, \end{array}$$

and

(26) wRIS=kmAkReCs+AkΔP−AoCb

for the resistance-in-series model, or

(27) wEPD=kmReΔP−AoCb−ApCs

for the effective pressure drop model. In (26) and (27), the dimensionless permeability $k_{m}$ is defined as

(28) $$\begin{array}{c} k_{m}=\frac{{\hat{K}}_{m}}{B\nu },\;k_{f}=\frac{{\hat{K}}_{f}}{B\nu }. \end{array}$$

A complete list of all boundary conditions is provided in Table 1. The dimensionless permeate flow rate is

(29) $$q=\frac{\int_{\Gamma_{n}}w_{i}dA}{\int_{\Gamma_{m}}dA},\;i=\left\{\mathrm{RIS},\;\mathrm{EPD}\right\}.$$

The 3D model (20)–(29) is implemented through the customized solver SUMs (Stanford University Membrane solver) in the open source finite volume simulator OpenFOAM, where an implicit time scheme for the transient solver and second order discretization in space are employed. The numerical mesh of the simulation is generated by a built-in OpenFOAM mesh tool, SnappyHexMesh, and the mesh resolution is determined such that the thinnest throat in the channel contains 15 numerical grids.

## 3. Experimental Data and Image Post-Processing

**Figure 2:**
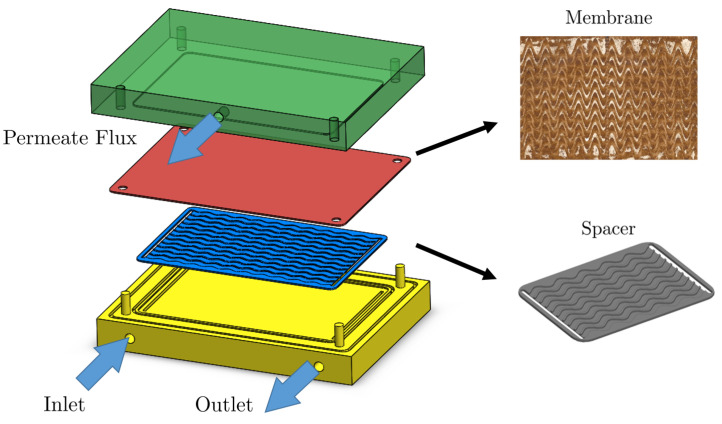
Three-dimensional rendering of the experimental setup by Xie et al. [24]. The membrane after flooding experiment together with a detailed rendering of spacer structure are shown on the right-hand side.

Experiments were performed with spacers inserted into a flat-sheet crossflow test cell. Each spacer formed ten equivalent flow paths on the membrane, see Figure 2. Each flow path was 6 mm wide and 1.5 mm high. The membrane was on the 6 mm side of the flow path. The straight-line distance between the entrance and exit of each flow path was 130 mm, resulting in an active membrane area of 780 mm^2^ for all configurations.

**Figure 3:**
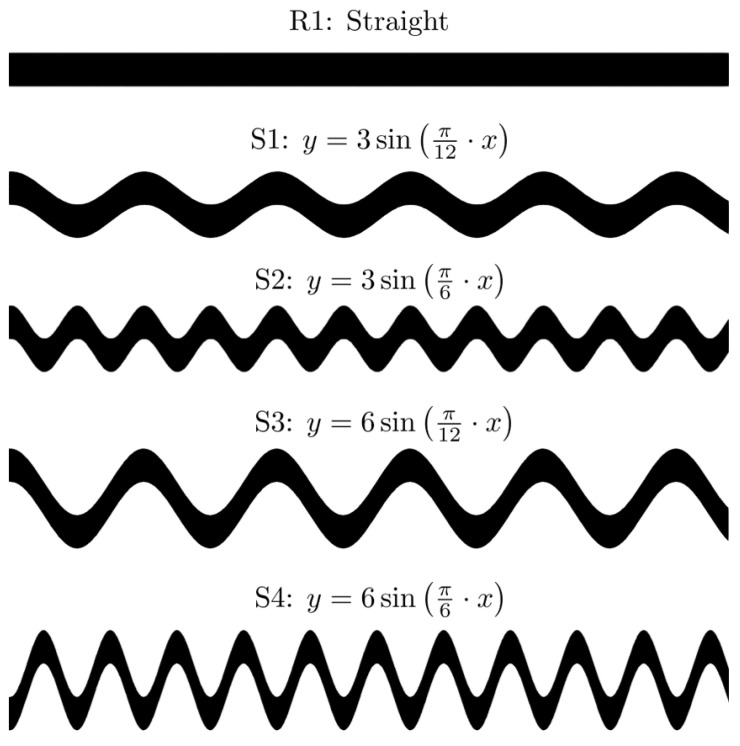
Spacer geometries R1, S1, S2, S3, and S4, and corresponding sinusoidal functions.

The experiments involved four sinusoidal spacers with different amplitudes and periods and a straight channel membrane for benchmark, see Figure 3. The data collected involve measurements of steady-state permeate flux and pressure drop [44], as well as spatial distribution of the foulant on the membrane surface after flooding 1L concentrated solution. The full description of the setup, data collection procedure, and data type can be found in [24,44]. A list of experimental parameters is provided in Table 2. The experimental data collected include measured permeate flux, pressure drop, and fouling pattern on the membrane surface.

**Figure 4:**
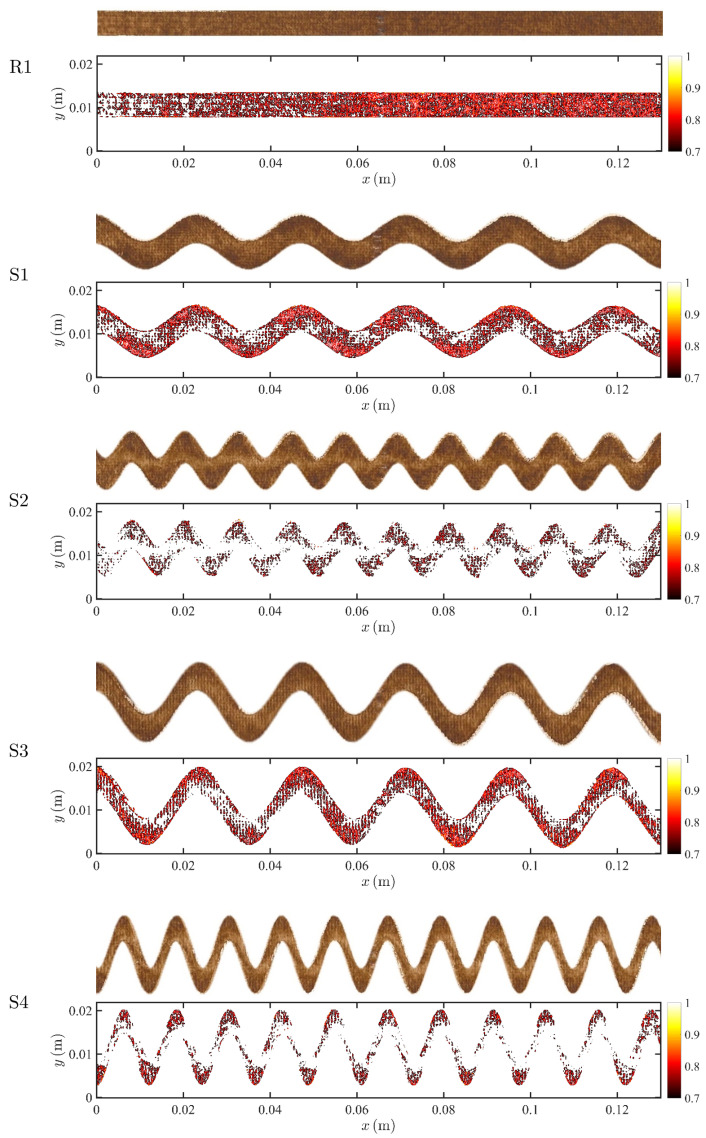
Images of the fouled membrane at the end of each experiment (top brown images) and digitalized fouling patterns (bottom red images) based on the gray scale of the experimental results. The threshold of all the images is set to be α=0.7.

Images of fouling patterns for different spacer morphologies need to be processed to map color intensity into surface concentration for comparison with numerical simulation. This is achieved in three sequential steps: (i) one flow channel is extracted from the raw image of the membrane, (ii) the image color intensity (in gray scale) is mapped to surface concentration according to

(30) $$\begin{array}{c} C_{s,\exp}=C_{s,\max}\cdot \frac{I}{I_{\max}}, \end{array}$$

where $C_{s,\exp}$ is the surface concentration from the experiment, $I_{\max}$ is the maximum gray scale intensity and *I* is the gray scale intensity at a given location; (iii) the experimental fouling patterns for the different spacers morphologies are obtained by thresholding the surface concentration as specified in Equation (17), i.e., surface concentration equal to or higher than the threshold value $\alpha C_{s,\max}$ (with α=0.7) is used to represent the experimental fouling pattern. In Figure 4, we show the unprocessed pictures of the fouling patterns in a single channel (top) and the fouling patterns after mapping to concentration fields (bottom) for each spacer morphology. The latter are used for a direct comparison with numerically simulated fouling patterns as discussed in the following section.

## 4. Results and Discussion

In this section, we present the simulation results from the two fouling models, the RIS and the EPD, defined by Equations (26) and (27), respectively. Both models are used to predict fouling, steady state permeate flux, and pressure drop for all five geometries.

The simulation parameters are set equal to the values reported in the experiments [24], and listed in Table 2. Additionally, studies on membrane adsorption/desorption rates have shown that the ratio between $K_{2}$ and $K_{1}$ varies from 0.001 to 1 [48]. In our study, we set $\theta =K_{2}/K_{1}=0.1$. More specifically, since the absolute values of $K_{1}$ and $K_{2}$ only affect how fast the foulant reaches equilibrium $C_{s,\max}$, we select $K_{1}=0.1$ and $K_{2}=\theta K_{1}=0.01$. The dimensionless number corresponding to the experimental conditions investigated are reported in Table 3, where Re is determined by the experimental parameters, and ${\mathrm{Da}}_{I}$ and ${\mathrm{Da}}_{\mathrm{II}}$ are determined by the selection of $K_{1}$ and $K_{2}$. Furthermore, $A_{o}$ is fitted by using the experimental data of R1. We note that, in addition to the parameters listed above, which are shared by both the RIS and EPD models, each model has one undetermined parameter: $A_{k}$ in the RIS model, and $A_{p}$ in the EPD model. Such parameters are fitted from experimental flux measurements on the benchmark rectangular geometry, R1, and then kept constant to predict flux, pressure, and fouling pattern for the all other geometries with $A_{k}=0.067$ and $A_{p}=3600$, for the RIS and EPD models, respectively. In each simulation, the inlet concentration is set to $C_{b}=1$ when $t=t_{0}$, i.e.,

(31) $$C_{b}{|}_{x\in \Gamma_{i}}=\left\{\begin{array}{cc} 0 & t<t_{0},\\ 1 & t⩾t_{0}. \end{array}\right.$$

For all simulations $t_{0}=120\;\left[s\right]$ and the total simulated time is 0.5 h.

### 4.1. Steady-State Flux and Pressure

**Figure 5:**
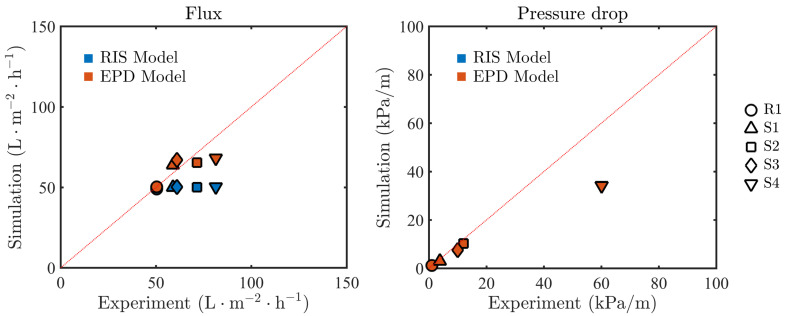
Comparison between the simulated permeate flux and pressure drop and the experimental results. Different shapes indicate results of different channel shapes, and the blue and red markers represent the RIS and the EPD models, respectively.

We compare the steady-state permeate flux measured in the experiments with the simulated flux. The flux is numerically computed from Equation (16) with $\hat{W}$ defined by (14) or (15) for the RIS and EPD models, respectively. The pressure drop, $\Delta {\hat{P}}_{L}$, is calculated as

(32) $$\Delta {\hat{P}}_{L}=\frac{{\hat{P}}_{\mathrm{in}}-{\hat{P}}_{\mathrm{out}}}{L},$$

where ${\hat{P}}_{\mathrm{in}}$ is the average pressure at the inlet, ${\hat{P}}_{\mathrm{out}}$ is the imposed pressure at the outlet, and *L* is the total length of the channel. In Figure 5, we plot both the measured and the simulated permeate fluxes from the RIS and EPD models, as well as the pressure drop for all five spacer configurations. The EPD model exhibits better agreement with the experimental flux than the RIS model, which underestimates the experimental flux when the geometry is more torturous (S1–S4). The difference in performance between the two models can be explained as follows. When the geometry is more torturous, the foulant distribution exhibits a more heterogeneous pattern along the membrane (see Figure 4), and it is associated with a less uniform velocity distribution. In the RIS model, the flow resistance is modeled by the combination of the membrane resistance and the foulant resistance, where the membrane permeability is a small value: as a result, the local flux is less sensitive to foulant distribution heterogeneity. On the other hand, in the EPD model, effective pressure loss due to the foulant is calculated by using a linear dependence which allows for accounting for the direct impact of local foulant variations on flux. Both models provided similar longitudinal pressure drop prediction, and the results are in good agreement with the experimental results, except for the S4 geometry. A good match with experiments is expected since the flux in RO systems is small compared to the crossflow velocity and thus flux boundary conditions would not significantly alter the longitudinal flow. Overall, the results suggest that, for the straight spacer (R1), both the RIS and EPD models can match the experimental results, while, for sinusoidal spacers (S1–S4), the EPD model can more accurately predict the measured flux. To estimate the overall accuracy of each model, we define error associated with the prediction of the permeate flux,

(33) $$\begin{array}{c} Err_{f}=\frac{\sqrt{\Sigma {\left[\left(q_{i,\mathrm{sim}}-q_{i,\exp}\right)/q_{i,\exp}\right]}^{2}}}{N}\;i=\left\{R1,S1–S4\right\}, \end{array}$$

where $q_{i,\mathrm{sim}}$ and $q_{i,\exp}$ are the numerical and experiment pressure drop, respectively. We also define the error associated with the pressure drop estimation as:

(34) $$\begin{array}{c} Err_{p}=\frac{\sqrt{\Sigma {\left[\left(\Delta {\hat{P}}_{i,\mathrm{sim}}-\Delta {\hat{P}}_{i,\exp}\right)/\Delta {\hat{P}}_{i,\exp}\right]}^{2}}}{N}\;i=\left\{R1,S1–S4\right\}, \end{array}$$

where $\Delta {\hat{P}}_{i,\mathrm{sim}}$ and $\Delta {\hat{P}}_{i,\exp}$ are the flux results of simulation and experiment, respectively. The error of the RIS is $Err_{f}=10.78\%$ and $Err_{p}=11.93\%$, the error of the EPD model is $Err_{f}=4.51\%$ and $Err_{p}=11.99\%$, which is consistent with Figure 5.

### 4.2. Dynamics

Tracking flux decline is an essential component of assessing the filtration process as the decline curve tracks the correlation between foulant accumulation and flux reduction.

**Figure 6:**
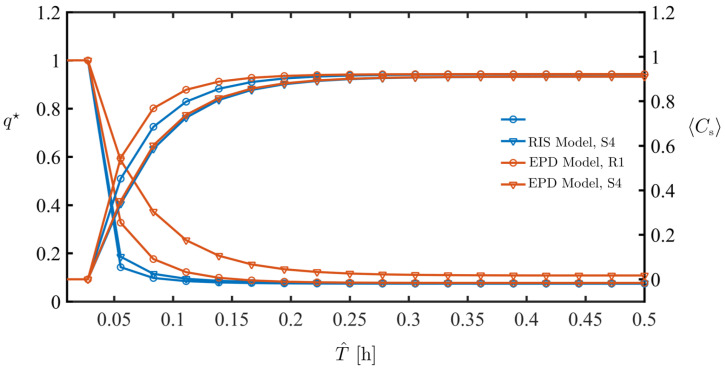
Comparison of the flux decline (left axis) of R1 and S4, and foulant accumulation (right axis) of R1 and S4 using RIS and EPD boundary conditions.

In Figure 6, we plot both the average permeate flux normalized by the flux before solute injection begins,

(35) $$\begin{array}{c} q^{🟉}=\frac{q}{q(t<t_{0})}, \end{array}$$

as well as the average foulant accumulation defined as

(36) $$\begin{array}{c} \langle C_{s}\rangle =\frac{\int_{\Gamma_{m}}C_{s}dA}{\int_{\Gamma_{m}}dA}, \end{array}$$

for the R1 and the S4 geometries and the RIS and EPD models. Both models show transient flux reduction and the flux results are closely coupled with foulant accumulation: as the foulant builds up, permeate flux decreases. Both models predict similar foulant accumulation, although the EPD model shows faster foulant buildup in the initial stage. For the flux reduction curves, the RIS shows little dependence on the two geometries, while the EPD model is able to better capture flux differences between between R1 and S4.

### 4.3. Fouling Pattern

Once the models have been validated against device-scale measurements, we proceed to test their ability to reproduce the spatial distribution of fouling patterns at steady state. For the RIS and the EPD models, we select the $\alpha =\alpha^{*}$ individually to plot the foulant distribution, where $\alpha^{*}$ is determined such that the foulant coverage reaches 50% of the total area of the membrane, i.e.,

(37) $$\begin{array}{c} \frac{\int_{\Gamma_{n}}dA}{\int_{\Gamma_{m}}dA}=0.5,\;\mathrm{where}\;\Gamma_{n}\in \left\{\Gamma_{m}|C_{s}\geq \alpha^{*}C_{s,\max}\right\} \end{array}$$

**Figure 7:**
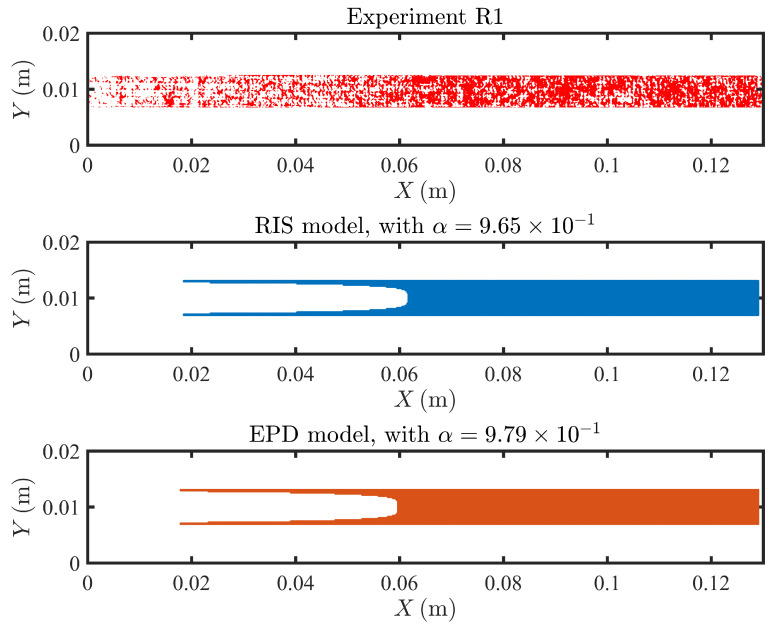
Comparison of the fouling pattern of R1, between the experimental results (in red), the RIS model simulation results (in blue) and the EPD model (in orange).

**Figure 8:**
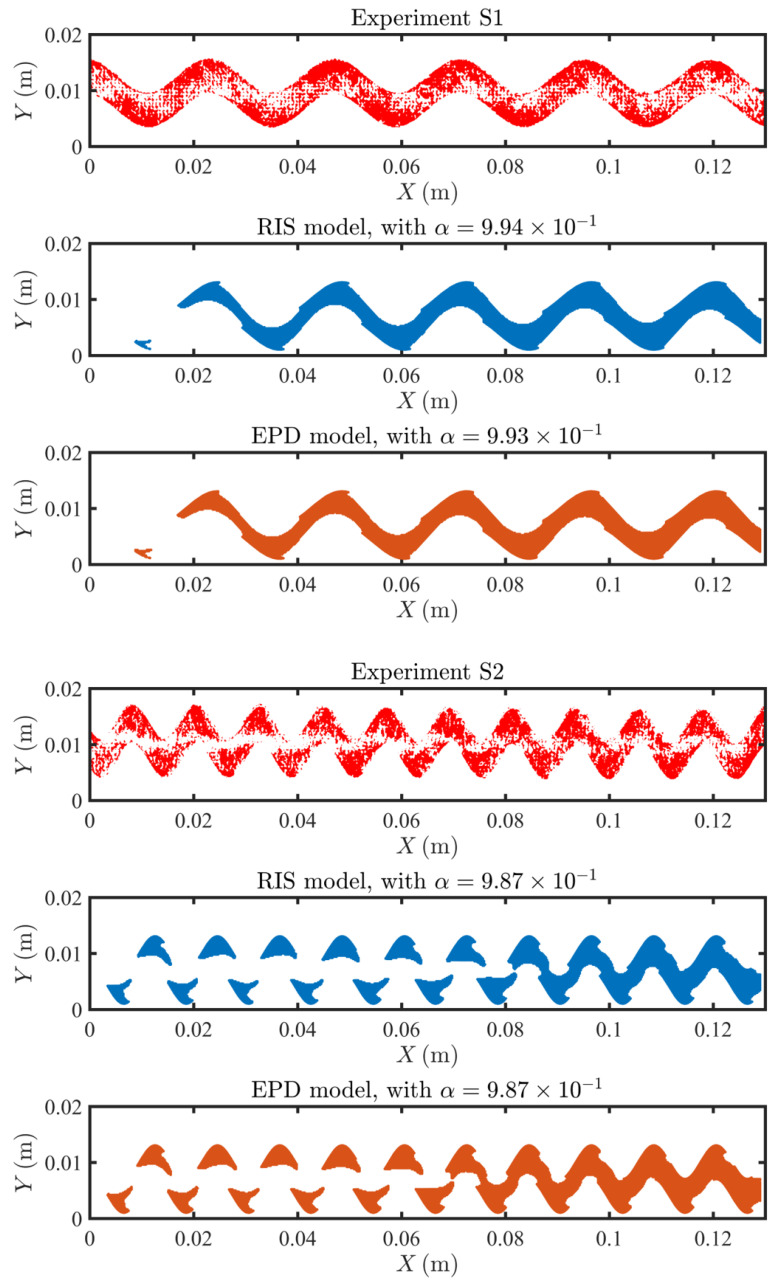
Comparison of the fouling pattern of S1 and S2, between the experimental results (in red), the RIS model simulation results (in blue) and the EPD model (in orange).

**Figure 9:**
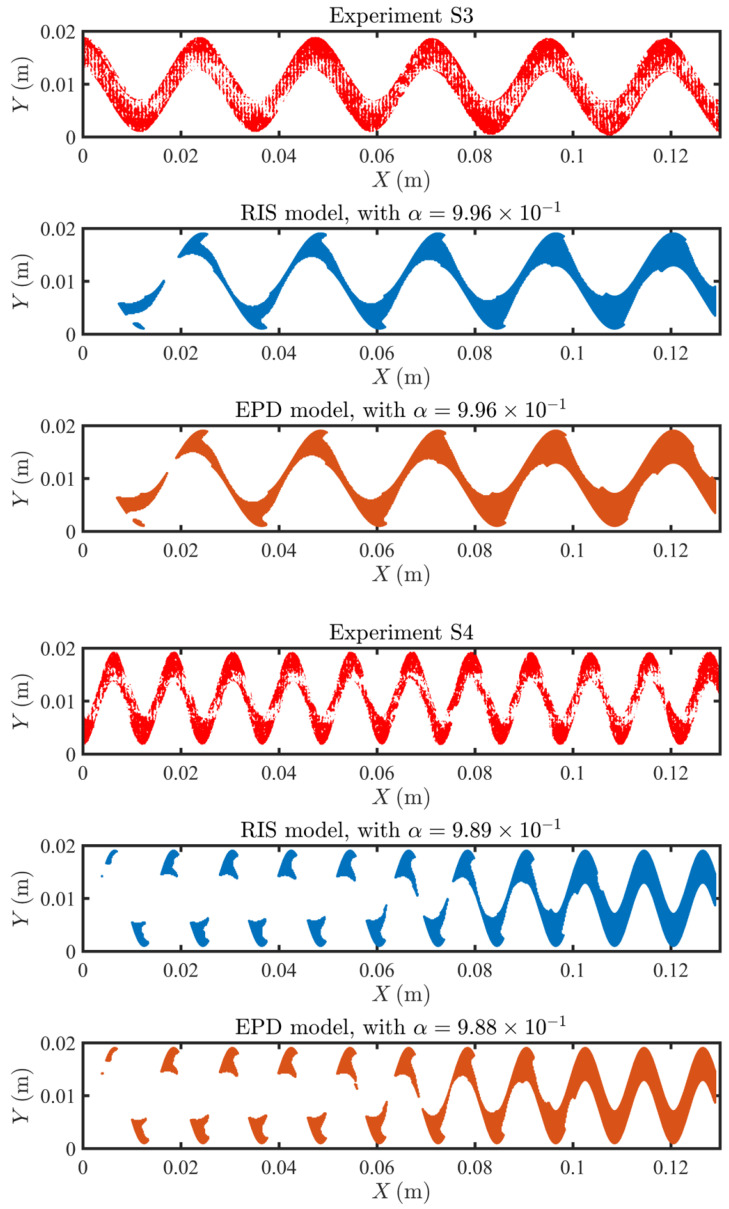
Comparison of the fouling pattern of S3 and S4, between the experimental results (in red), the RIS model simulation results (in blue) and the EPD model (in orange).

In Figure 7, Figure 8 and Figure 9, we compare the experimental and predicted fouling patterns from the two models for all geometries. Overall, the model results show good agreement with data regardless of the flux boundary condition used. Specifically, the models correctly capture a number of features in the experimental fouling patterns: (i) more foulant accumulates near the outlet than at the inlet, (ii) foulant accumulates at the peaks and troughs of the sinusoidal channel, and (iii) for sinusoidal spacers with larger amplitude, the fouling pattern develops an asymmetric shape with not symmetric tails extending upstream.

Overall, the fouling pattern exhibits strong spatial heterogeneity, a result of coupling between adsorption and local flow conditions, which can significantly differ across channel morphologies. A framework coupling between flow, solute transport, and foulant accumulation is robust in modeling heterogeneous spacers and can accurately predict high fouling zones.

## 5. Conclusions

In this study, we investigate the ability of two different fouling models (RIS and EPD) to correctly capture both system-scale performance quantities, namely permeate flux and pressure drop, as well as fine-scale features, such as high fouling regions. The two models are constructed as boundary conditions on the membrane surface and implemented in the code SUMs within the OpenFOAM framework. Both fouling models have only one fitting parameter, calibrated against the rectangular membrane benchmark geometry. Fit-free predictions are then performed on four membranes with sinusoidal spacers with different amplitudes and frequencies. Model predictions are tested against the experimental data, which included both system scale measurements (flux decline curves and pressure drop) and local measurements (fouling patterns). Both models were overall able to capture both (i) system-scale pressure drop and (ii) spatio-temporal fouling patterns for five different spacer geometries, although the EPD model was more sensitive to the impact of spacer morphologies on flux, and therefore better able to predict both flux decline and steady-state flux for different morphologies. Both the RIS and the EPD models were successful in capturing the spatial distribution of foulant, and its main experimentally observed features. These results suggest that such a framework is able to successfully (i) simulate flow, transport, and fouling process using transient equations; (ii) couple the flow, bulk concentration and surface concentration of the foulant dynamically while elucidating foulant accumulation mechanisms; (iii) associate concentration polarization with fouling by using an adsorption type equation, and (iv) incorporate different flux reduction models such as the RIS model and the EPD model. Future work includes generalization of the code to Membrane Distillation (MD) processes and other filtration techniques as well as to more complex spacer geometries and system-scale (i.e., module scale) domains. Moreover, as a three-dimensional simulator, SUMs are compatible with three-dimensional geometries imported directly from design tools. As a result, the code can be directly used for filtration systems optimization in industrial applications.

## Figures and Tables

**Table 1:** Boundary Conditions for the simulation.

Boundary	Flow	Bulk Concentration	Pressure
	u	$C_{b}$	P
Inlet, $\Gamma_{i}$	u=(1,0,0)	$C_{b}=1$	∂P/∂n=0
Outlet, $\Gamma_{o}$	∂u/∂n=0	$\partial C_{b}/\partial n=0$	∂P/∂n=0
Solid Wall, $\Gamma_{w}$	u=0	$\partial C_{b}/\partial n=0$	∂P/∂n=0
Membrane, $\Gamma_{m}$	$u=(u_{h},v_{h},w_{h})$	$\partial C_{b}/\partial n=w_{h}C_{b}$	${\hat{P}}_{\mathrm{out}}/\left(\nu^{2}/B^{2}\right)$

**Table 2:** Experimental parameters, symbols, values, and corresponding units.

Parameter	Experimental Parameters		
	Symbol	Value	[L,T,M]
Width	*B*	$6\times 10^{-3}$	m
Height	*H*	$1.5\times 10^{-3}$	m
Viscosity	ν	$1\times 10^{-6}$	$m^{2}/s$
Diffusion Coeff.	*D*	$2\times 10^{-11}$	$m^{2}/s$
Outlet Pressure	$P_{\mathrm{out}}$	4137	Psi
Inlet Velocity	$U_{\mathrm{in}}$	0.15	m/s
Concentration	$C_{0}$	50	mmol/L
Permeability	$K_{m}$	$5\times 10^{-8}$	m/(s·kPa)

**Table 3:** The fixed parameters of all the simulations.

Model	*Re*	${\mathrm{Da}}_{I}$	${\mathrm{Da}}_{\mathrm{II}}$	$A_{o}$	β	$A_{k}$	$A_{p}$
RIS EPD	900	$5\times 10^{7}$	$4\times 10^{-3}$	0.067	2	0.07 -	- 3600

## Data Availability

Not applicable.

## Nomenclature

Abbreviation
RIS	resistance-in-series
EPD	effective-pressure-drop
SUMs	Stanford University Membrane solver
Parameters
$\hat{T}$	time
$\hat{X}$	location vector
$\hat{X},\hat{Y},\hat{Z}$	location coordinate
*B*	spacer width
*H*	spacer height
*L*	spacer length
$\hat{U}$	velocity
$\hat{U},\hat{V},\hat{W}$	velocity components
$\hat{P}$	pressure
ρ	density
ν	kinematic viscosity
${\hat{C}}_{b}$	bulk concentration
*D*	molecular diffusion coefficient
$K_{1}$	adsorption coefficient
$K_{2}$	desorption coefficient
${\hat{C}}_{s,\max}$	equilibrium foulant concentration
$\hat{\beta }$	porous media structure parameter
$\hat{W}$	permeate water flux
$R_{i}$	intrinsic membrane rejection rate
${\hat{K}}_{m}$	hydraulic membrane water permeability
$\Delta \hat{P}$	pressure drop
$\hat{P}$	local pressure
$P_{\mathrm{amb}}$	ambient pressure
${\hat{P}}_{\mathrm{in}}$	average inlet pressure
${\hat{P}}_{\mathrm{out}}$	outlet pressure
${\hat{A}}_{o}$	osmotic coefficient
${\hat{K}}_{\mathrm{eff}}$	fouled membrane permeability
$R_{m}$	clean membrane resistance
$R_{f}$	is the fouled membrane resistance
${\hat{A}}_{p}$	foulant coefficient
$\hat{Q}$	permeate flow rate
$\Gamma_{n}$	non-fouled region
Re	Reynolds
Pe	Péclet
${\mathrm{Da}}_{i}$	Damköhler number
$Err_{f}$	error of flux
$Err_{p}$	error of pressure
